# Nonsyndromic bilateral and unilateral optic nerve aplasia: first familial occurrence and potential implication of *CYP26A1* and *CYP26C1* genes

**Published:** 2011-08-05

**Authors:** Françoise Meire, Isabelle Delpierre, Cecile Brachet, Françoise Roulez, Christian Van Nechel, Fanny Depasse, Catherine Christophe, Björn Menten, Elfride De Baere

**Affiliations:** 1 Department of Ophthalmology, Queen Fabiola Children's University Hospital, Brussels, Belgium; 2 Department of Radiology, Queen Fabiola Children's University Hospital, Brussels, Belgium; 3 Pediatric Endocrinology, Queen Fabiola Children's University Hospital, Brussels, Belgium; 4 Department of Neurophysiology, Erasme Hospital, Brussels, Belgium; 5 Center for Medical Genetics, Ghent University Hospital, Ghent, Belgium

## Abstract

**Purpose:**

Optic nerve aplasia (ONA, OMIM 165550) is a very rare unilateral or bilateral condition that leads to blindness in the affected eye, and is usually associated with other ocular abnormalities. Although bilateral ONA often occurs in association with severe congenital anomalies of the brain, nonsyndromic sporadic forms with bilateral ONA have been described. So far, no autosomal-dominant nonsyndromic ONA has been reported. The genetic basis of this condition remains largely unknown, as no developmental genes other than paired box gene 6 (*PAX6*) are known to be implicated in sporadic bilateral ONA.

**Methods:**

The individuals reported underwent extensive ophthalmological, endocrinological, and neurologic evaluation, including neuroimaging of the visual pathways. In addition genomewide copy number screening was performed.

**Results:**

Here we report an autosomal-dominant form of nonsyndromic ONA in a Belgian pedigree, with unilateral microphthalmia and ONA in the second generation (II:1), and bilateral ONA in two sibs of the third generation (III:1; III:2). No *PAX6* mutation was found. Genome wide copy number screening revealed a microdeletion of maximal 363 kb of chromosome 10q23.33q23.33 in all affected individuals (II:1, III:1; III:2) and in unaffected I:1, containing three genes: exocyst complex component 6 (*EXOC6*), cytochrome p450, subfamily XXVIA, polypeptide 1 (*CYP26A1*), and cytochrome p450, subfamily XXVIC, polypeptide 1 (*CYP26C1*). The latter two encode retinoic acid-degrading enzymes.

**Conclusions:**

This is the first study reporting an autosomal-dominant form of nonsyndromic ONA. The diagnostic value of neuroimaging in uncovering ONA in microphthalmic patients is demonstrated. Although involvement of other genetic factors cannot be ruled out, our study might point to a role of *CYP26A1* and *CYP26C1* in the pathogenesis of nonsyndromic ONA.

## Introduction

Optic nerve aplasia (ONA, OMIM #165550) is a very rare congenital anomaly that can be unilateral or bilateral. ONA is usually associated with other ocular abnormalities such as punched-out chorioretinal defects, retinal dysplasia, coloboma, microphthalmos, cataracts, and sclerocornea [1]. There may be a scleral aperture, but there are no retinal vessels. ONA always causes total blindness of the affected eye. The disorder is ophthalmoscopically distinct from optic nerve hypoplasia. Taylor et al. [1] introduced diagnostic criteria and a classification of ONA. Bilateral cases often occur in association with severe congenital anomalies of the brain [2]; however, unilateral and bilateral ONA have been reported in otherwise healthy children [3-5]. Apart from two sisters with putative nonsyndromic bilateral aplasia described by Newman and coworkers in 1864 [1], all nonsyndromic reported cases with bilateral ONA have been sporadic so far ( [1] and references therein).

The genetic basis of ONA is largely unknown. A paired box gene 6 (*PAX6*) missense mutation, p.T391A, has been described in a patient with bilateral ONA, nystagmus, and normal anterior eye segments. Apart from absence of the optic nerves, no other abnormalities were observed in this patient, who exhibited normal growth, a normal physical exam, and karyotype [6].

Here we report, for the first time, the familial occurrence of nonsyndromic ONA in a father and his dizygotic twins. Their phenotypes are documented with ophthalmological, endocrinological, and neurologic evaluation, including neuroimaging of the visual pathways. Genomewide copy number screening using microarray-based comparative genomic hybridization (arrayCGH) revealed a microdeletion of 10q23.33q23.33, potentially implicating the cytochrome p450, subfamily XXVIA, polypeptide 1 (*CYP26A1*) and cytochrome p450, subfamily XXVIC, polypeptide 1 (*CYP26C1*) genes encoding retinoic acid (RA)-degrading enzymes as novel candidate genes for ONA.

## Methods

### Patients

For this study, we enrolled a consenting family with a healthy grandmother (I:1), an affected father (II:1), his affected twins (III:1, III:2), and his unaffected partner (II:2). The couple was Caucasian and nonconsanguineous. There were no other children. Family history was negative. The study was conducted following the tenets of Helsinki and was approved by our local Institutional Review Boards.

### Clinical evaluation

The ophthalmological evaluation consisted of fundoscopy, ultrasound, Doppler examination, and visual evoked potentials (VEP). Endocrinological evaluation was performed as follows: target height range was calculated as (father’s height + mother’s height±13 cm)/2±8.5 cm [7]. Birthweight and birth length data are expressed as standard deviation scores (SDS) using the Niklasson references [8]. Height was measured using a Harpender stadiometer and data are expressed as SDS using the Cole references [9]. Laboratory investigations included growth hormone (GH) stimulation test, insulin-like growth factor 1 (IGF1), prolactin, and thyroid function measurements, antiendomysium antibodies, and plasma and urine osmolarity. Neuroimaging was performed using magnetic resonance imaging (MRI) of the brain and orbits in II:1, III:1, and III:2.

### Genetic testing

Genomic DNA was extracted from leukocytes using the Puregene (Gentra, Qiagen, Venlo, The Netherlands) and QiaAmp DNA isolation kit (Qiagen, Venlo, The Netherlands). Sequencing of coding exons of *PAX6,* orthodenticle, drosophila, homolog of, 2 (*OTX2*), and SRY-box 2 (*SOX2*) was performed as described [10-12]. All family members underwent genome-wide copy number screening with 60 K Agilent oligonucleotide arrays as described (Agilent Technologies, Diegem, Belgium) [13]. Hybridizations were performed according to the manufacturer's instructions with minor modifications. The results were subsequently visualized in arrayCGHbase [14].

## Results

A couple with dizygotic twins with blindness due to bilateral ONA was admitted for genetic counseling. The father wore a scleral prosthesis on his left microphthalmic eye. The family history was unremarkable otherwise. The couple requested a second opinion about the recurrence risk for ONA.

Twin pregnancy was obtained after intracytoplasmic sperm injection. Intake of thyroxine during gestation was reported in the context of maternal Hashimoto thyroiditis (chronic lymphocytic thyroiditis). The girl (III:2) had a birthweight of 2,150 g (−2.1 standard deviation score [SDS]) and a length of 43 cm (−2.6 SDS) [8]. The boy (III:1) had a birthweight of 2,320 g (−1.9 SDS) and a length of 46 cm (−1.4 SDS). Both children required nasogastric feeding in the neonatal period. At the age of three weeks, blindness was suspected in both children and confirmed by ophthalmologic examination. Karyotyping was normal.

The children were three years old when first examined by us. Both children showed normal neurodevelopmental milestones, taking into account their blindness [15]. Development of language and performance skills was normal for the age.

### Ophthalmologic examination

**Figure 1:**
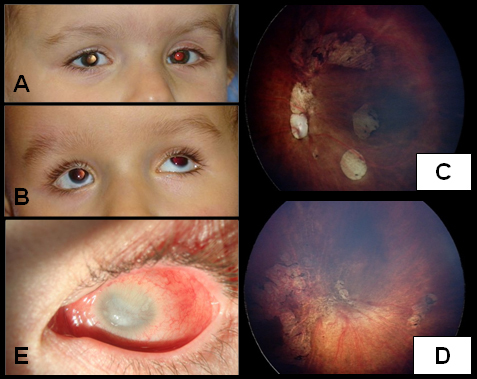
Clinical pictures of III:2, III:1 and II:1. **A**: A picture of III:2 with mild bilateral microphthalmia with 10.5 mm corneal diameters and atypical coloboma of the iris in the right eye. **B**: A picture of twin brother III:1 with bilateral microphthalmia with 9 mm corneal diameters and scleralization of the inferior cornea. **C**-**D**: Fundus picture of III:1 showing the absence of the optic nerve, dysplastic retinae, and a few retinal vessels. **E**: A picture of the father’s (II:1) left eye, showing unilateral left microphthalmos (corneal diameter of 7 mm) with a vascularized cornea, impairing the view to the anterior segment and to the fundus.

Both children (III:1 and III:2) had no light perception. III:2 presented with mild bilateral microphthalmia with 10.5 mm corneal diameters and atypical coloboma of the iris in the right eye (Figure 1A). The pupils were nonreactive to light. Lenses were transparent. Fundoscopy revealed absence of the optic nerves and retinal vasculature in both eyes. The presence of retinal dysplasia was observed. There was no associated chorioretinal coloboma.

The twin brother (III:1) of child III:2 presented bilateral microphthalmia with 9 mm corneal diameters and scleralization of the inferior cornea (Figure 1B). The pupils were round and nonreactive to light. Ophthalmoscopic examination disclosed the absence of the optic nerve, dysplastic retinae, and a few retinal vessels (Figure 1C,D).

**Figure 2:**
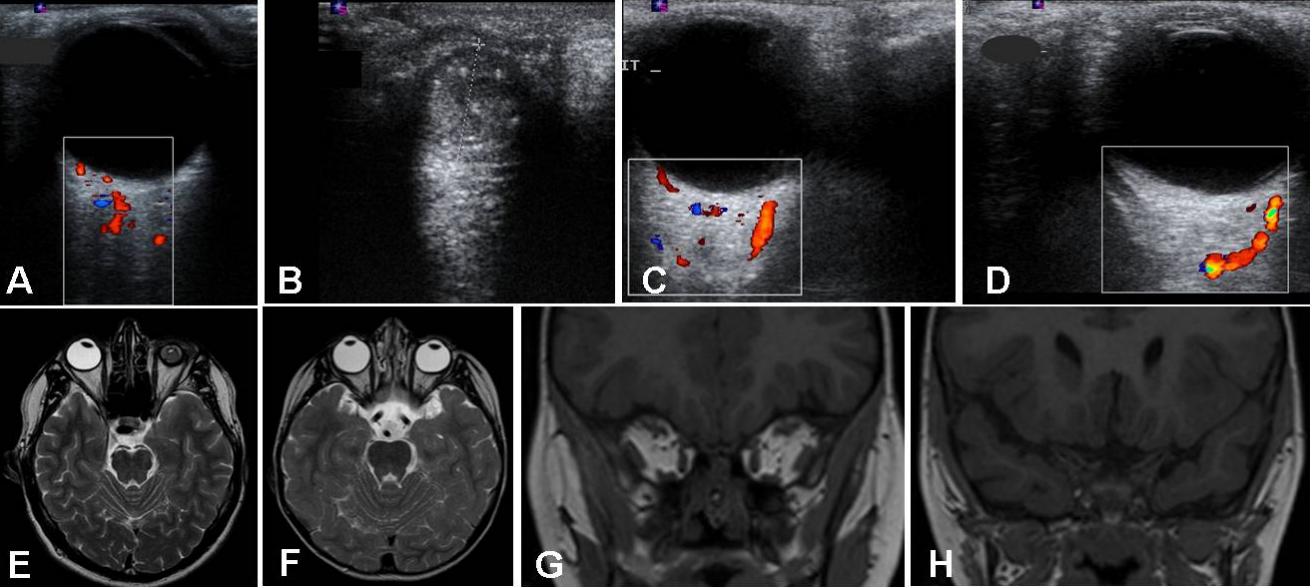
Ultrasound and MRI findings in III:1 and II:1. The father’s (II:1) Doppler ultrasonographic examination. This demonstrates **A**: a normal right eye with the optic nerve and *arteria centralis retinae*; **B**: a left microphthalmic heterogeneous eye without the optic nerve visible. **C, D**: The son’s (III:1) Doppler ultrasonographic examination demonstrating the absence of both optic nerves and corresponding vascularization, but the presence of posterior ciliary vessels (**C** and **D** for right and left eye, respectively). The vessels are represented in color. The Doppler examination is represented in the boxes. **E**: II:1’s axial T2-weighted image in MRI demonstrated a normal right eye and lens and a left microphthalmic eye with thick sclera. **F**: III:1’s axial T2-weighted image in MRI showed an almost normal morphology of both eyes but the absence of optic nerves (with some remnants of dural sheath), chiasms, and tracts. **G**-**H**: Coronal T1-weighted MRI images in the midorbital (**G**) and intracranial (**H**) planes. This shows the complete lack of both orbital nerves in the son, III:1.

Examination of the father (II:1) revealed unilateral left microphthalmos (corneal diameter of 7 mm) with vascularized cornea, impairing the view to the anterior segment and to the fundus (Figure 1E). Doppler ultrasonography of II:1 showed a normal right eye, with normal optic nerve and *arteria centralis retinae* (Figure 2A), and a left microphthalmos with an axial length of 14.7 mm, a cataractous lens, and absence of the optic nerve (Figure 2B).

Doppler ultrasonography of the eye and orbit in III:1 showed a normal structure in both eyes, with a slight reduction in the anteroposterior size of the left eye (19.9 mm) compared to the right one (21.7 mm). It also showed complete absence of both optic nerves and corresponding vascularization (Figure 2C,D). Doppler examination revealed the presence of a few blood vessels entering the posterior pole and distributed in an irregular pattern (Figure 2C,D).

VEPs in II:1 were registered after pattern reversal full-field stimulation of the right eye. The symmetry of the distribution of responses over both hemispheres was analyzed. Normal responses were registered in the left hemisphere, but with a larger amplitude of P100.

Clinical ophthalmological assessment of the grandmother (I:1; best corrected visual acuity, slit lamp examination, fundoscopy) revealed no abnormalities.

### Endocrinological assessment

The father’s (II:1) height was 169 cm, and the mother’s (II:2) height was 164.7 cm. Midparental target height was 160.9±8.5 cm for girls and 173.4 ±8.5 cm for boys.

III:2 had a birthweight of 2,150 g (−2.1 SDS) and a birth length of 43 cm (−2.6 SDS). At the age of three years and seven months, her physical examination showed: standing height 88.9 cm (−2.5 SDS), weight 10.5 kg, body mass index 13.3 kg/m^2^ (−2.3 SDS), and head circumference 48 cm (−1.9 SDS). The growth curve showed relatively regular growth. Bone age was three years according to Greulich and Pyle [16]. Laboratory investigations showed normal IGF1 (107 ng/ml), normal thyroid function tests and prolactin, and negative antiendomysium antibodies. A GH stimulation test (glucagon) showed a normal GH peak value (34.2 ng/ml, n>10) and a normal cortisol response.

III:1 had a birthweight of 2,320 g (−1.9 SDS) and a birth length of 46 cm (−1.4 SDS). At the age of three years and seven months, his physical examination showed: standing height 90 cm (−2.5 SDS), weight 11 kg, body mass index 13.6 kg/m^2^ (−2.2 SDS), and head circumference 48.7 cm (−2.3 SDS). The growth curve showed relatively regular growth. Bone age was two years and eight months according to Greulich and Pyle [16]. Laboratory investigations showed normal IGF1 (111 ng/ml), normal thyroid function tests and prolactin, and negative antiendomysium antibodies. A GH stimulation test (glucagon) showed a GH peak value of 5.2 ng/ml, (n>10) and a normal cortisol response. Fasting plasma osmolarity was 285 mOSm/kg H_2_O and fasting urine osmolarity was 885 mOSm/kg H_2_O, which demonstrates a normal urine-concentrating ability.

### Neuroimaging

Brain and orbit MRI in II:1 (Figure 2E) confirmed normal morphology and size of the right eye, lens, and nerve, and absence of optic nerve (orbital and prechiasmatic part) in the left eye. The chiasm was asymmetric. The optic tract size was asymmetric, the left being larger than the right. The left orbit was microphthalmic with a thick sclera. Brain MRI was normal.

Brain and orbit MRI examination of both children (III:1 and III:2) confirmed a normal aspect in both eyes with a slight reduction in size of the globe. Both optic nerves, both tracts, and the chiasm were absent (Figure 2F-H) in each child. The anatomies of the brain and pituitary gland were normal.

### Genetic study

**Figure 3:**
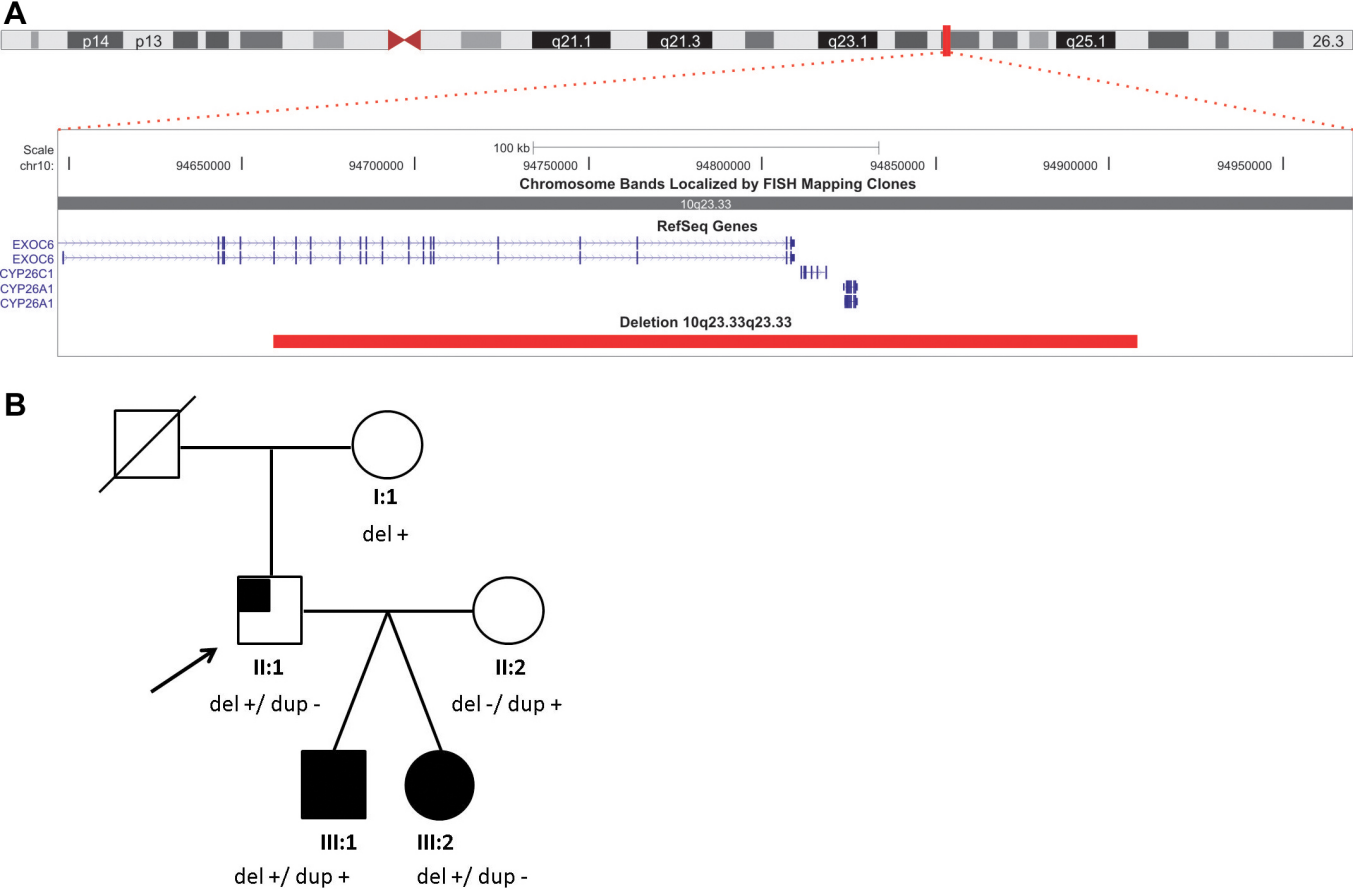
UCSC track of the 10q microdeletion. **A**: The 10q23.33q23.33 track shows the extent of the249–363 kb deletion of chromosome band 10q23.33q23.33 (arr 10q23.33q23.33(94659243–94908060)x1 pat 10q23.33q23.33. The location and size of the deletion are indicated by a horizontal red bar. The figure was drawn according to the UCSC, Human Genome Browser, March 2006 (NCBI36/hg18). **B**: The three-generation pedigree represents the unaffected grandmother (I:1), affected father (II:1), and twins (III:1 and III:2) carrying the 10q23 deletion. Del: deletion of 10q23.33q23.33; dup: duplication of 2p16.2p16.2. Filled symbol: bilateral optic nerve aplasia (ONA). Partially filled symbol: unilateral ONA.

Mutation screening in three developmental genes—*PAX6, OTX2,* and *SOX2*—revealed no pathogenic mutations. Genome-wide microarray-based comparative genome hybridization (arrayCGH) in III:1 revealed an abnormal male arrayCGH profile: a 249–363 kb deletion of chromosome band 10q23.33q23.33 and an 86–215 kb duplication of chromosome band 2p16.2p16.2 (Figure 3A). The 10q23.33q23.33 deletion was also found in his twin sister (III:2) and father (II:1). The duplication was also found in II:2, and was absent in II:1 and III:2 (Figure 3B). The deletion was present in the unaffected grandmother (I:1). The deleted region contains three genes: exocyst complex component 6 (*EXOC6*), cytochrome p450, subfamily XXVIA, polypeptide 1 (*CYP26A1*), and and cytochrome p450, subfamily XXVIC, polypeptide 1 (*CYP26C1*; Figure 3A). Apart from those copy number variations (CNVs), no other CNVs were found in the proband III:1. No other deletions of this region are present in our local patient database (~2,000 patients, ~2,000 controls).

## Discussion

Newman et al. [17] reported on two blind sisters with absent optic discs and retinal vessels. However, there exists some doubt about the true nature of ONA in these siblings [17]. Moreover, autosomal recessive inheritance cannot be excluded. Apart from this family, nonsyndromic ONA has never been reported in a familial context. Here, we report autosomal-dominant nonsyndromic ONA in a father and his dizygotic twins for the first time.

The histopathology of eyes with ONA has been described previously [18], reporting the absence of ganglion cells, optic nerve fibers, and retinal vessels. The retinal pigment epithelium covered the area where the optic disc should have been [19], and remnants of the dural sheath were identified. The *arteria centralis retinae* was lacking, although the existence of a few rudimentary retinal vessels entering the posterior pole in a chaotic way has been reported [20]. Doppler examination in our patients clearly demonstrated the absence of an *areteria centralis retinae,* but some ciliary vasculature was present entering the posterior pole. Neovascularization in ONA has been reported in neonates with subsequent tractional retinal detachment [21]. Life-long risk for choroidal neovascularization exists, and has been well documented by Pieramici et al. [22].

The incidence of optic aplasia in microphthalmic eyes has never been studied. Diagnosis of ONA in the microphthalmic eye of II:1 was performed by MRI imaging, as fundoscopy was not possible because the eye was severely microphthalmic with an opaque cornea. This illustrates that ONA may remain underdiagnosed in severely microphthalmic eyes. Therefore, MRI imaging in microphthalmos is recommended to exclude ONA.

In addition, MRI of the brain is essential to diagnose associated malformations of the central nervous system. The association of hypopituitarism and severe microphthalmos and anophthalmos, as well as the association of congenital hypopituitarism with ONA, have been reported [23-25].

In these twins with ONA (III:1 and III:2) the hypothalamic-pituitary function seemed normal: growth was regular; no episodes of hypoglycemia had been noted; free T4 levels, IGF1, and cortisol plasma levels were normal; and there was no diabetes insipidus. GH peak values were normal in the girl and subnormal in the boy, but the GH stimulation tests have a low positive predictive value [26,27]. Anatomically, the hypothalamic-pituitary axis was normal on MRI. The short stature probably resulted from intrauterine growth retardation.

Neuroimaging of the visual pathways in the twins (III:1 and III:2) showed absence of chiasm and tractus. Imaging of the visual pathways in the father with left ONA proved a complete absence of the left optic nerve, although with optic tract asymmetry. VEP findings were correlated with this MRI observation. VEP in the father demonstrated the existence of contralateral crossing nerve fibers, and hence a functional posterior pathway contralaterally. Moreover, stimulation of the normal eye resulted in a response that had a larger amplitude on the contralateral cortex than on the ipsilateral cortex, suggesting abnormal nerve crossing, with more nerve fibers crossing on the chiasm. Misrouting of nerve fibers of the normal eye in unilateral ONA may be the result of nonmeeting retinal axons from the side with ONA.

The pathogenesis of ONA is unknown. The fact that eyes with optic aplasia may be nearly normal in size and have a normal lens suggests normal initial development of the eye with primitive multipotent retinal ganglion cells with vascular supply from both the hyaloid artery and the annular vessel. At six weeks post conception, the optic stalk is almost completely filled by nerve fibers. At three months, axons of ganglion cells pass through the glial lamina cribrosa at the optic nerve head. During the second month, a primitive vascular network in the mesenchyme around the optic cup (annular vessel) and precursors of the posterior ciliary arteries that arose from the ophthalmic artery connect and form the precursor of the choroidal vasculature [28]. This choroidal vasculature is normal in ONA. Primitive retinal vessels emerge early in the fourth month from cell clusters near the hyaloid artery as it enters the optic disc. These buds then push into the nerve fiber layer, and the proximal intraneural portion of the hyaloid vessels becomes the central retinal artery and vein. The observation of the absence of retinal vessels and lacunar retinal defects in ONA might suggest that defective retinal development and failure of retinal angiogenesis in the third to fourth month may contribute to the degeneration of retinal ganglion cells [28]. Defective retinal angiogenesis and retinal dysplasia in ONA could be associated with coloboma of the eyes.

Both environmental and genetic factors are hypothesized to contribute to unilateral ONA. Here, unilateral and bilateral ONA occur in the same family in an autosomal-dominant fashion, assuming a genetic basis. So far, only mutations in the developmental genes *PAX6* and *OTX2* have been reported in ONA [6]. Mutation screening of these genes, however, was negative in this family. A microdeletion of 10q23.33q23.33 was found in the affected father and his affected children, but also in the unaffected grandmother. The paternal grandfather is deceased and the father has no siblings. The deletion contained three known genes: *EXOC6, CYP26A1* and *CYP26C1*. In the Toronto database, two copy number variations of the *EXOC6* gene have been reported in control individuals [29,30]. While CNVs (i.e., gains) containing *EXOC6* have been reported in two control individuals, no CNVs of *CYP26A1* and *CYP26C1* have been described so far. Interestingly, the latter two encode retinoic acid-degrading enzymes. Although a long-range effect of the deletion on neighboring upstream or downstream genes cannot be excluded, we might postulate that haploinsufficiency of the *CYP26A1* and *CYP26C1* genes is causally related to the ONA phenotype in this family. Of note, two cases with partial trisomy of 10q24.1-ter with concomitant 7pter and 4qter deletion share ONA and malformation of the anterior chamber [31]. As no fine-mapping was performed of the 10q24 breakpoints at that time, involvement of the *CYP26A1* and *CYP26C1* genes cannot be excluded. *CYP26A1* and *CYP26C1*, encoding RA-degrading enzymes, might be interesting candidate genes contributing to the pathogenesis of optic nerve defects when mutated. CYP26 enzymes are thought to play a central role in appropriate regulation of the RA signal as a posteriorizing factor in central nervous system development [32-34]. Mice and humans possess three *CYP26* genes: *CYP26A1, CYP26B1*, and *CYP26C1* [35-37].

The functions of Cyp26a1 and Cyp26c1 have been studied in knockout mice. Loss of *Cyp26c1* did not appear to affect embryonic development, suggesting that *Cyp26a1* and *Cyp26c1* are functionally redundant. Studies in mice lacking both genes suggested that the activity of *Cyp26a1* and *Cyp26c1* is required for correct anteroposterior patterning and the production of migratory cranial neural crest cells in the developing mammalian brain [38]. Importantly, *Cyp26* expression is known to be more distinctive during the later stages of retina formation in mice [38]. The presence of retinal dysplasia in the family studied here might be attributed to defective embryogenesis.

The absence of any ocular abnormalities in a carrier of the deletion (I:1) might suggest reduced penetrance. This might be attributed to the redundancy of the *CYP26B1* gene, or to modifier effects and environmental factors influencing RA metabolism, resulting in an overall *CYP26* expression above the threshold, and hence normal RA metabolism in I:1. An alternative explanation might be somatic mosaicism of an as yet unidentified genetic defect in the father (II:1).

The role of additional environmental factors such as intracytoplasmic sperm injection in the more severe, bilateral phenotypic expression in the twins is unclear at this moment [39,40]. In-depth studies of the retinal morphology of *Cyp26* knockout mice or other model organisms with knockdowns of *Cyp26a1* and *Cyp26c1* will be instrumental to understanding their role in the pathogenesis of ONA.

### Conclusion

This is the first study reporting an autosomal-dominant form of nonsyndromic unilateral and bilateral ONA. We demonstrated that neuroimaging (e.g., MRI) may have an important diagnostic value for uncovering ONA in microphthalmic patients. Finally, our findings implicate the deletion of the *CYP26A1* and *CYP26C1* genes as potential susceptibility factor for ONA.
